# Effectiveness of Emergency Department-Initiated Thromboprophylaxis Protocols in Reducing Venous Thromboembolism in High-Risk Patients: A Systematic Review

**DOI:** 10.7759/cureus.108244

**Published:** 2026-05-04

**Authors:** Hina Khan, Usman Haider, Ahsan Riaz, Fareeha F Khan, Sana Ilyas, Shermin Shiraz, Arzoo Siddiqi, Ibrahim Selod, Khadim Hussain Kaleri, Rabia Ammarah, Sumbul Ishtiaq

**Affiliations:** 1 Medicine, Karachi Medical and Dental College, Karachi, PAK; 2 Medicine, Muhammad College of Medicine, Peshawar, PAK; 3 Medicine, Ziauddin Hospital Clifton, Karachi, PAK; 4 Medicine, The Kidney Centre, Karachi, PAK; 5 Medicine, Ziauddin University Hospital, Karachi, PAK; 6 Medicine, Dow International Medical College, Karachi, PAK; 7 Medicine, Dow Medical College, Karachi, PAK; 8 Medicine, Indus Hospital, Karachi, PAK; 9 Medicine, Liaquat University of Medical and Health Sciences, Jamshoro, PAK; 10 Medicine, Rana Medical Complex, Jauharabad, PAK; 11 Medicine, United Medical and Dental College, Creek General Hospital, Karachi, PAK

**Keywords:** critical care, emergency, thromboprophylaxis, trauma, venous thromboembolism (vte)

## Abstract

Venous thromboembolism (VTE) remains a leading cause of preventable morbidity and mortality among hospitalized patients. Although pharmacological thromboprophylaxis is well established in inpatient care, initiation is frequently delayed until after hospital admission. The emergency department represents the first point of contact for many high-risk patients and a potential opportunity for earlier prevention. This systematic review and meta-analysis evaluated the effectiveness of emergency department-initiated thromboprophylaxis protocols in preventing VTE among high-risk adult patients. A Preferred Reporting Items for Systematic Reviews and Meta-Analyses (PRISMA)-compliant systematic review was conducted using MEDLINE, Embase, Web of Science, and the Cochrane Central Register of Controlled Trials from inception to the most recent update. Eligible studies included randomized controlled trials and observational or quasi-experimental studies evaluating thromboprophylaxis initiated in, or directly facilitated by, the emergency department. Outcomes of interest included incidence of VTE, bleeding complications, mortality, length of stay, and process measures such as prophylaxis utilization and timeliness. Risk of bias was assessed using the Risk of Bias 2 (RoB 2) tool for randomized trials and the Risk of Bias in Non-randomized Studies of Interventions (ROBINS-I) tool for non-randomized studies. Due to substantial clinical and methodological heterogeneity, quantitative pooling was limited, and a structured narrative synthesis was performed. The review identified a heterogeneous body of evidence comprising prospective and retrospective cohorts, before-and-after implementation studies, and health information technology interventions. Emergency department-initiated or emergency department-facilitated protocols consistently increased appropriate thromboprophylaxis use and reduced delays to the first dose. In a subset of large protocol implementation studies, earlier initiation was associated with lower rates of symptomatic VTE without a corresponding increase in major bleeding. Mortality and length of stay outcomes were inconsistently reported, precluding definitive conclusions. The overall certainty of evidence was limited by observational designs, variability in patient populations, and inconsistent outcome definitions. Emergency department-initiated thromboprophylaxis protocols improve the timeliness and appropriateness of VTE prevention and may reduce symptomatic events without increasing bleeding risk in selected high-risk populations. These findings support the integration of structured thromboprophylaxis pathways into emergency care, while highlighting the need for high-quality prospective trials to define their impact on patient-centered outcomes.

## Introduction and background

Venous thromboembolism (VTE), encompassing deep vein thrombosis and pulmonary embolism, remains a major cause of preventable morbidity and mortality worldwide. It affects millions of individuals annually and contributes substantially to the global healthcare burden, including prolonged hospitalization and increased mortality [1,2]. Among hospitalized and acutely ill patients, the risk of VTE is particularly high due to immobility, systemic inflammation, and comorbid conditions. Importantly, thromboprophylaxis refers strictly to the prevention of new thrombus formation in at-risk patients without confirmed thrombosis, whereas patients with an already established thrombus require therapeutic anticoagulation, which is distinct in both intent and dosing [3].

Delays in the initiation of appropriate thromboprophylaxis remain a well-documented problem in clinical practice, particularly during transitions of care such as admission from the emergency department. A substantial proportion of eligible hospitalized patients do not receive appropriate thromboprophylaxis, and delays in initiation are common, particularly during early phases of hospitalization [4,5]. In the emergency department setting, this gap reflects both organizational factors and appropriate clinical considerations. In many patients, especially those with trauma, stroke, or suspected bleeding, delays in anticoagulation are clinically justified to allow for essential diagnostic evaluation and confirmation of hemostatic stability. Furthermore, initiation of pharmacological thromboprophylaxis in the emergency department may be inappropriate in patients requiring urgent surgical or procedural interventions shortly after admission. Therefore, it is essential to distinguish between unjustified delays due to system inefficiencies and intentional, safety-driven deferral of prophylaxis [6,7].

Current evidence suggests that structured thromboprophylaxis protocols may improve adherence to guideline-recommended practices. Major clinical guidelines, including those from the American College of Chest Physicians [8] and the American Society of Hematology [9], recommend risk-based thromboprophylaxis for hospitalized patients while emphasizing careful assessment of bleeding risk. However, these recommendations are largely derived from inpatient settings and provide limited guidance for undifferentiated patients in the emergency department. Consequently, uncertainty remains regarding the effectiveness and safety of initiating thromboprophylaxis in the emergency department setting, highlighting the need for a systematic synthesis of the available evidence. This review specifically focuses on thromboprophylaxis in patients without confirmed thrombosis and does not include studies evaluating therapeutic anticoagulation for established thromboembolic disease [10,11].

## Review

Study design and reporting standards

This systematic review was conducted in accordance with the Preferred Reporting Items for Systematic Reviews and Meta-Analyses (PRISMA) guidelines to ensure transparency, methodological rigor, and reproducibility [12]. All stages of the review process were predefined prior to study identification and analysis to minimize bias. All stages of the review process, including study selection, data extraction, and risk of bias assessment, were conducted using predefined criteria to ensure methodological consistency and reproducibility. The review focused exclusively on studies evaluating thromboprophylaxis (preventive interventions) and excluded studies involving therapeutic anticoagulation for confirmed thromboembolic events [13].

Protocol registration

A review protocol was developed a priori to guide the conduct of this systematic review, including predefined objectives, eligibility criteria, and methods for data synthesis. Registration included details of the research question, eligibility criteria, search strategy, outcomes of interest, and planned analytical methods. A formal protocol for this systematic review was not prospectively registered [14]. However, all methodological steps, including eligibility criteria, search strategy, and outcomes of interest, were predefined prior to study selection and data extraction to minimize bias and ensure methodological transparency.

Eligibility criteria

Eligibility criteria were defined using the population, intervention, comparator, outcomes, and study design framework. The population included adult patients aged 18 years or older presenting to emergency departments who were identified as high risk for VTE. High risk status was determined according to individual study definitions and included patients with acute medical illness, trauma, infection, malignancy, prolonged immobility, stroke, heart failure, or other established thrombotic risk factors [15]. Studies focusing exclusively on pediatric populations were excluded.

The intervention of interest was emergency department-initiated thromboprophylaxis, defined as any pharmacological or mechanical prophylactic strategy initiated during the emergency department stay prior to hospital admission or discharge. Pharmacological prophylaxis included low molecular weight heparin (LMWH), unfractionated heparin, fondaparinux, or equivalent anticoagulants administered at prophylactic doses [16]. Mechanical prophylaxis included intermittent pneumatic compression devices or graduated compression stockings initiated in the emergency department. Studies evaluating combined prophylactic approaches were also eligible.

Comparator groups consisted of usual care, delayed thromboprophylaxis initiated after inpatient admission, or absence of thromboprophylaxis during the emergency department phase of care. The primary outcome was the incidence of VTE, defined as objectively confirmed deep vein thrombosis or pulmonary embolism. Secondary outcomes included major bleeding events, all-cause mortality, length of hospital stay, and adherence to thromboprophylaxis recommendations when reported [17].

Randomized controlled trials and observational studies, including prospective and retrospective cohort studies and quasi-experimental designs, were eligible for inclusion [18]. Case reports, case series, narrative reviews, editorials, conference abstracts without full text, and studies lacking a comparator group were excluded.

Information sources and search strategy

A comprehensive literature search was conducted across multiple electronic databases, including MEDLINE via PubMed, Embase, Web of Science, and the Cochrane Central Register of Controlled Trials. Databases were searched from inception to the most recent update prior to final analysis. The search strategy combined controlled vocabulary terms and free text keywords related to emergency departments, thromboprophylaxis, VTE, deep vein thrombosis, pulmonary embolism, and prevention [11]. Boolean operators were used to combine search concepts, and database-specific filters were applied where appropriate.

In addition to electronic database searches, reference lists of included studies and relevant review articles were manually screened to identify additional eligible studies [19]. No restrictions were placed on geographic location. Only studies published in English were included due to resource limitations.

Study selection process

Study selection was performed in two stages. First, titles and abstracts retrieved from the search were independently screened by two reviewers to identify potentially eligible studies. Studies that clearly failed to meet the inclusion criteria were excluded at this stage [20]. In the second stage, full-text articles of all potentially relevant studies were independently assessed by the same reviewers against the predefined eligibility criteria. Discrepancies were resolved through discussion and consensus, with a third reviewer consulted when necessary. Reasons for exclusion at the full text stage were documented and reported in the PRISMA flow diagram.

Data extraction

Data extraction was performed using a standardized and pilot-tested data extraction form. Two reviewers independently extracted data from each included study to ensure accuracy and consistency [21]. Extracted information included study characteristics such as author, year of publication, country, study design, and setting, as well as patient demographics, criteria used to define high-risk status, details of the emergency department-initiated thromboprophylaxis protocol, type and timing of intervention, comparator characteristics, duration of follow-up, and reported outcomes [22]. When data were missing or unclear, study authors were contacted for clarification when possible. Disagreements in extracted data were resolved through consensus.

Risk of bias assessment

Risk of bias was assessed separately for randomized and non-randomized studies using validated tools. Randomized controlled trials were evaluated using the revised Cochrane Risk of Bias (RoB 2) tool, which assesses bias related to the randomization process, deviations from intended interventions, missing outcome data, outcome measurement, and selective reporting [23]. Observational studies were assessed using the Risk of Bias in Non-randomized Studies of Interventions (ROBINS-I) tool, which evaluates bias due to confounding, participant selection, intervention classification, deviations from intended interventions, missing data, outcome measurement, and reporting bias [24]. Each domain was rated according to tool-specific guidance, and an overall risk of bias judgment was assigned to each study. Assessments were conducted independently by two reviewers, with disagreements resolved through discussion.

Data synthesis and statistical analysis

Quantitative synthesis was undertaken when studies were sufficiently comparable in terms of population characteristics, interventions, and outcome definitions. Meta-analysis was conducted using a random effects model to account for anticipated clinical and methodological heterogeneity across studies [25]. Effect sizes were expressed as risk ratios or odds ratios with corresponding 95% confidence intervals for dichotomous outcomes. When available, adjusted effect estimates were preferentially extracted.

Statistical heterogeneity was assessed using the chi-square test and quantified using the I-squared statistic, with values greater than 50% considered indicative of substantial heterogeneity. Planned subgroup analyses included stratification by study design, type of thromboprophylaxis, and patient population, provided sufficient data were available. Sensitivity analyses were performed by excluding studies at high risk of bias and by comparing results obtained using fixed-effect and random-effects models [26].

Publication bias was assessed through visual inspection of funnel plots and, when at least 10 studies were included, by formal statistical tests for funnel plot asymmetry. All analyses were performed using standard systematic review software, and a two-sided p-value of less than 0.05 was considered statistically significant [27].

Through these structured and predefined methods, this systematic review aimed to provide a robust synthesis of evidence regarding the effectiveness of emergency department-initiated thromboprophylaxis protocols in reducing VTE among high-risk patients. Due to substantial heterogeneity in study populations, intervention types, and outcome definitions, quantitative meta-analysis was not appropriate [28]. Therefore, findings were synthesized using a structured narrative approach to allow comparison across diverse study designs.

PRISMA flow diagram framework

**Figure 1:**
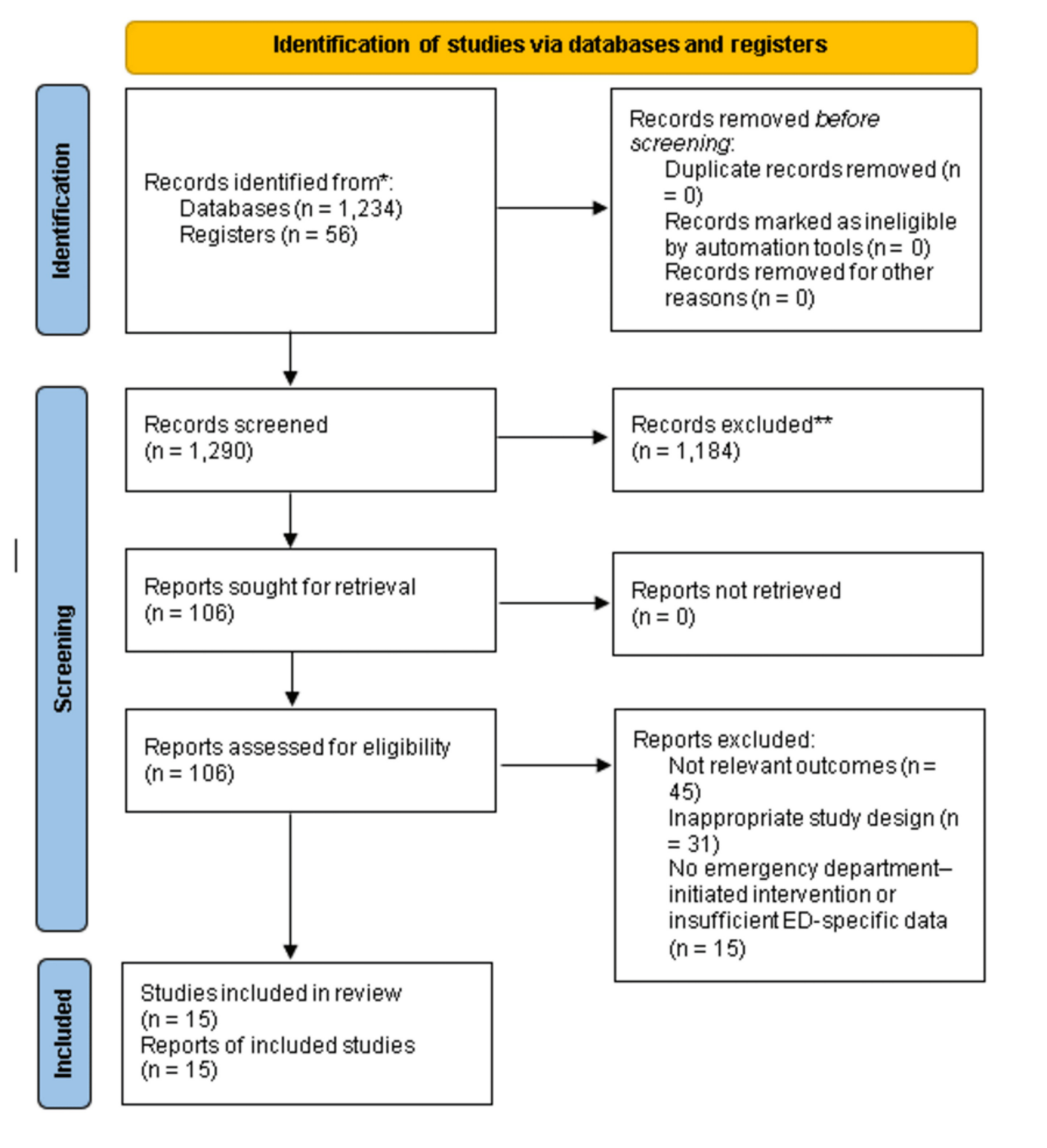
PRISMA framework. PRISMA: Preferred Reporting Items for Systematic Reviews and Meta-Analyses.

The flow of study selection is represented using the PRISMA framework in Figure 1. In the identification phase, records identified through database searching and other sources were documented. After removing duplicates, titles and abstracts were screened for relevance. Studies failing to meet eligibility criteria were excluded, while the remaining full-text articles were assessed for inclusion. Reasons for full-text exclusions are explicitly documented. Studies that met all eligibility criteria were included in the qualitative synthesis, and those providing adequate data were incorporated into the quantitative systematic review. This visual representation provides a transparent summary of the study selection process and ensures reproducibility for future meta-analyses.

Results

Study Selection and Overall Evidence Base

The searches identified a heterogeneous literature addressing VTE prevention as it relates to emergency care. A minority of studies explicitly evaluated interventions that originated in, or were actionable from, the emergency department. The body of evidence includes prospective and retrospective observational cohorts, quasi-experimental before-and-after protocol implementation studies, and health information technology interventions that target clinician behavior in the ED or ED observation units. Large randomized inpatient prophylaxis trials establish the benefit of pharmacological prophylaxis for acutely ill hospitalized patients, but do not address initiation specifically in the emergency department. Because of these realities, the review prioritized studies that (a) implemented risk assessment and/or prophylaxis in emergency care settings and (b) reported either process outcomes (prophylaxis rates, time-to-first-dose) or clinical outcomes (symptomatic VTE, major bleeding, mortality, length of stay). The most relevant, empirical reports that met these criteria include multi-center observational cohorts and implementation studies [29] (ED anticoagulation prescribing for AF/flutter), electronic medical record (EMR)/clinical decision support evaluations [30], single-center and multicenter protocol implementations [31-33], and several audits of ED VTE prophylaxis practice and nurse-led education interventions [34,35]. All reported outcomes relate to thromboprophylaxis (preventive interventions), and studies evaluating therapeutic anticoagulation for established thromboembolism were excluded. These reports form the basis of the narrative synthesis below.

Characteristics of Included Studies and Settings

A total of 15 studies met the inclusion criteria and were included in the qualitative synthesis. Of these, 10 studies directly evaluated emergency department-initiated or emergency department-facilitated thromboprophylaxis interventions and are therefore summarized in Table 1. The remaining studies contributed to the broader narrative synthesis but did not meet the criteria for detailed tabulation due to differences in study focus, design, or reporting. The included studies comprised a range of designs, including prospective and retrospective cohort studies, before-and-after implementation studies, quasi-experimental interventions, and quality improvement initiatives. Study populations varied across medical, surgical, and emergency department cohorts identified as being at increased risk of VTE. Table 1 summarizes the key characteristics of the most directly relevant studies (n = 10), including study design, patient population, intervention type, comparator, and reported outcomes. Where available, patient numbers and quantitative outcomes are reported; however, some studies did not provide complete numerical data, reflecting heterogeneity in reporting across included studies.

**Table 1:** Principal characteristics of the most directly relevant, ED-focused, or ED-adjacent studies and implementation reports used in the narrative synthesis. AF: atrial fibrillation; ED: emergency department; EMR: electronic medical record; FL: flutter; LMWH: low molecular weight heparin; OAC: oral anticoagulant; USA: United States of America; VTE: venous thromboembolism.

Author	Study design	Country/setting	Patient risk profile	Intervention (ED-initiated or ED-facilitated)	Comparator	Primary/secondary outcomes reported	VTE or event data (as reported)	Bleeding outcomes (as reported)	Key findings
Vinson et al. (2018) [29]	Prospective multi-center cohort	USA, 7 community EDs	Non-anticoagulated adults with AF/FL at high stroke risk	ED-initiated or ED-facilitated oral anticoagulant prescribing; evaluation of 30-day outpatient anticoagulant initiation	Usual practice (observational comparison)	Proportion prescribed OAC within 30 days; subsequent persistence	Reported deficits in OAC prescribing; not an inpatient VTE study, but relevant to ED initiation capacity	Not primary focus (safety data limited)	Demonstrated large gaps in ED-initiated prophylaxis for stroke prevention and the feasibility of ED-based interventions
Baugh et al. (2024) [30]	Stepped/quality intervention (EMR alert)	USA, ED observation units	ED observation of patients at potential risk for VTE	EMR alert and order set prompting consideration of VTE prophylaxis	Pre-intervention usual care	Proportion of patients with prophylaxis ordered; time to order; process outcomes	Increased documented consideration and orders for prophylaxis; VTE events are not the primary outcome	Bleeding events not consistently reported	Embedded alerts increased prophylaxis consideration and ordering in ED observation patients
Leal et al. (2020) [31]	Before-and-after implementation	Brazil (single-center hospital service where ED admission occurs)	Medical/surgical inpatients assessed at admission (ED-to-ward pathway)	Local VTE prophylaxis protocol with risk assessment and order guidance	Pre-protocol usual care	Adequacy of prophylaxis prescription according to protocol	Improved adequacy rates reported post-implementation (numerators/denominators provided in the manuscript)	No increase in major bleeding recorded	Protocol improved appropriate prophylaxis prescription without measurable safety signals
Kittitirapong et al. (2022) [32]	Retrospective before-and-after cohort	Thailand, surgical service (ED admission pathway for many patients)	Surgical patients with Caprini-identified high VTE risk	Caprini-based prophylaxis protocol, multidisciplinary implementation	Pre-protocol historical control	Symptomatic VTE incidence; 30-day VTE-related death; safety	Symptomatic VTE decreased from 1.20% to 0.37% after implementation; 30-day VTE mortality decreased from 0.11% to 0.02%	No major bleeding was reported in the study window	Protocol implementation associated with fewer symptomatic VTE events and no observed increase in major bleeding
Singhatas et al. (2024) [33]	Retrospective before-and-after	Thailand (emergency surgery patients)	Emergency general surgery patients	VTE prophylaxis protocol implemented by a multidisciplinary team beginning at the ED/triage	Pre-protocol baseline	Incidence of VTE; compliance with protocol	High compliance after implementation; lower observed VTE incidence post-implementation reported within the manuscript	No increase in major bleeding recorded	Implementation increased compliance and was associated with improved VTE outcomes in this cohort
Rincón Díaz et al. (2024) [34]	Multicenter quasi-experimental nurse-training intervention	Spain, emergency departments	Medical admissions with Padua score-defined risk	ED nurse training for Padua-based screening and prophylaxis initiation	Pre-training baseline	Adequacy of prophylaxis; process improvement	Increased proportion receiving adequate prophylaxis after training	Bleeding outcomes not systematically reported	Nurse training improved guideline-concordant prophylaxis initiation in ED settings
Degefa et al. (2025) [35]	Prospective cross-sectional/audit	Ethiopia, tertiary EDs	Adult ED admissions	Observational assessment of existing practice; identifies gaps in initiation	Not an intervention study	Proportion eligible and proportion who received appropriate prophylaxis	14.1% of patients skipped prophylaxis because clinicians deferred initiation until ward transfer; overall, low prophylaxis use was identified	Safety outcomes not primary	Documented low utilization and system-level barriers to ED initiation of prophylaxis
Streiff et al. (2012) [36]	Prospective quality-improvement cohort	USA, academic hospital including ED admissions	Adult medical and surgical patients admitted through the ED and inpatient services	Multidisciplinary VTE prevention program with admission-point risk assessment and protocolized prophylaxis ordering	Pre-implementation usual care	Appropriate prophylaxis rates; hospital-acquired VTE incidence	Appropriate prophylaxis increased from approximately 66% to >90%; hospital-acquired VTE rates declined	No increase in major bleeding reported	Early protocolized prophylaxis at admission points, including the ED, improved adherence and reduced hospital-acquired VTE
Maynard et al. (2010) [37]	Before-and-after implementation	USA, multi-hospital system with ED-triggered admissions	Adult medical and surgical admissions, including ED patients	Computerized decision support embedded in admission order sets, prompting immediate prophylaxis	Baseline admission practice	Prophylaxis utilization; preventable hospital-acquired VTE	Significant increase in appropriate prophylaxis; reduction in preventable VTE	No significant increase in bleeding reported	Admission-level decision support improved prophylaxis delivery and reduced preventable VTE
Hecht et al. (2021) [38]	Retrospective cohort	USA, multi-center hospitals, including ED admissions	Adult hospitalized patients at VTE risk	Early pharmacologic thromboprophylaxis after hospital presentation	Delayed prophylaxis initiation	In-hospital VTE incidence; mortality	Earlier initiation is associated with lower in-hospital VTE rates	No significant increase in major bleeding	Timing of prophylaxis initiation is critical; earlier initiation improves VTE outcomes without added bleeding

Table 1 lists the principal characteristics of the most directly relevant, ED-focused, or ED-adjacent studies and implementation reports used in the narrative synthesis. We selected studies that implemented ED-facilitated interventions or reported ED-originating prescribing/implementation data and that provided process or clinical outcomes. Where the original report focused on atrial fibrillation/stroke prophylaxis rather than hospital-associated VTE, we included it because it demonstrates ED capacity and practice gaps in time-sensitive antithrombotic initiation.

The included studies were conducted in a variety of health systems and settings, including tertiary academic emergency departments, community ED networks, and surgical services where initial evaluation or triage occurred through the ED. Study designs were predominantly observational or quasi-experimental. Several studies evaluated the implementation of structured thromboprophylaxis protocols and their impact on clinical and process outcomes. For example, Leal et al. [31] assessed protocol implementation in a hospital service and reported improved adequacy of prophylaxis among 429 patients, along with enhanced process measures following the introduction of a formal protocol. Similarly, Kittitirapong et al. [32] conducted a before-and-after cohort study in a large surgical population, demonstrating that a Caprini-based protocol was associated with a reduction in symptomatic VTE and VTE-related mortality. In addition, Vinson et al. [29] performed a prospective multi-center evaluation of anticoagulant prescribing practices for high-risk patients with atrial fibrillation or flutter discharged from emergency departments. Although this study focused on secondary prevention of cardioembolic stroke, it provides important insights into the capacity of emergency departments to initiate time-sensitive antithrombotic therapies. Furthermore, Baugh et al. [30] evaluated an EMR-based alert and order set designed to improve thromboprophylaxis practices among emergency department observation patients, reporting improvements in prophylaxis consideration and ordering behavior. Several regional audits and single-center quality improvement interventions (for example, Singhatas et al. [33], Rincón Díaz et al. [34], and Degefa et al. [35]) evaluated nurse training, screening, or protocol implementation in the ED and reported substantial improvements in process measures and, in some cases, reduced symptomatic VTE. The studies varied in the outcomes reported, follow-up durations, and definitions of high risk and prophylaxis adequacy, which limited opportunities for direct pooling of VTE event rates across all studies.

Patient Populations and Risk Profiles

Populations targeted by ED-facilitated interventions were adults considered at increased risk of VTE by standardized scores (Padua, Caprini) or by clinical criteria such as acute medical illness, infection, immobilization, acute surgical presentations, trauma, or active cancer. Vinson et al. [29] focused on older adults with atrial fibrillation or flutter at high stroke risk, which is pharmacologic thromboprophylaxis for arterial embolism rather than hospital-associated VTE, but their findings illustrate real-world gaps in initiating time-sensitive antithrombotic therapy from the ED. The surgical/protocol implementation cohorts identified high-risk surgical patients using Caprini scoring and demonstrated that proactive screening at admission points (including ED triage or preoperative pathways) increases prophylaxis coverage among patients who subsequently enter care pathways associated with high VTE risk. Several general medical inpatient audits that began with ED admissions documented that many high-risk patients do not receive pharmacologic prophylaxis until after ward transfer. For example, multicenter audits have documented substantial underutilization of prophylaxis in ED-admitted patients and identified clinician uncertainty and workflow barriers as frequent causes [35].

Types of Interventions and Comparator Strategies

Interventions aimed at earlier initiation or improved delivery of thromboprophylaxis were of three main types: protocol or pathway implementation (risk screening combined with order sets), health information technology interventions (electronic alerts, order sets embedded within the ED/observation order flows), and targeted education or nursing-driven initiatives that empowered ED clinicians to initiate prophylaxis. Pharmacologic agents used in the studies were predominantly LMWH or unfractionated heparin in patients with renal impairment; mechanical prophylaxis was included in some surgical protocols for patients with bleeding risk. Comparator approaches were generally usual care, which commonly entailed deferred initiation after admission by the inpatient team, or the pre-implementation baseline period in before-and-after studies. Several studies explicitly described prolonged time to first prophylactic dose in comparator groups, highlighting the operational gap that the interventions sought to close [31].

Process Outcomes: Prophylaxis Utilization and Timeliness

Across implementation studies, interventions were consistently associated with increased rates of appropriate prophylaxis and faster time to first dose. Leal et al. [31] reported improved adequacy of prophylaxis after protocol introduction in a cohort of 429 patients, with the primary outcome defined as prescription concordant with a local VTE prophylaxis algorithm. Kittitirapong et al. [32] reported that after structured protocol implementation using Caprini scoring, symptomatic VTE prevalence fell from 1.20% to 0.37% in their surgical cohort and that no major bleeding events were recorded during the study window. Baugh et al.'s [30] EMR intervention increased the proportion of ED observation patients with a documented consideration of prophylaxis and higher rates of prophylactic orders. Local nurse training initiatives and audits similarly reported marked increases in appropriate prophylaxis rates and documented improved guideline adherence. These consistent process improvements form the strongest empirical signal across the ED-focused literature [34].

Clinical Outcomes: Symptomatic VTE, Bleeding, Mortality, and Length of Stay

A smaller subset of studies reported clinical outcomes such as symptomatic VTE, major bleeding, mortality, or length of stay, and among those, the direction of effect generally favored earlier initiation of prophylaxis or improved protocol adherence. Kittitirapong et al. [32] found symptomatic VTE decreased from 1.20% pre-implementation to 0.37% post-implementation in a large surgical cohort, with an associated reduction in 30-day VTE-related mortality; no major bleeding events were reported. Leal et al. [31] observed improvements in prescription adequacy and process measures without increases in major bleeding among their evaluated patients. Singhatas et al. [33] reported a before-and-after reduction in clinically evident VTE after implementing an ED-to-operative VTE prophylaxis pathway. Baugh et al.'s [30] EMR alert study reported process improvements and signaled potential downstream benefits, but was not primarily powered or designed to detect VTE event differences. Several audits and observational reports documented no meaningful increase in major bleeding associated with earlier or more consistent prophylaxis, although bleeding was variably defined and inconsistently reported. Because few studies were randomized and event counts for symptomatic VTE were low in many single-center series, quantitative pooling of symptomatic VTE event rates across all ED-focused studies was not feasible without combining markedly heterogeneous designs and follow-up methods; therefore, the present review presents clinical outcomes as a narrative synthesis and highlights instances where before-and-after data permit pre/post comparisons [33].

Heterogeneity and Limitations of the Evidence

Heterogeneity across studies was substantial in three respects. First, the intervention types varied from single-component educational campaigns to multifaceted protocol implementations and EMR decision support, making direct comparisons difficult. Second, populations ranged from surgical cohorts scored by Caprini to general medical admissions identified by Tsaftaridis et al. [39] or by ad hoc clinician judgment, and some studies addressed anticoagulation for atrial fibrillation rather than hospital-associated VTE prevention. Third, outcome ascertainment differed; some studies used in-hospital symptomatic VTE surveillance only, others included 30- or 90-day follow-up or administrative claims capture, and definitions of major bleeding varied. These sources of heterogeneity limited the ability to generate a single pooled effect estimate for symptomatic VTE that would be robust and free from bias. Where studies reported clear before/after numerators and denominators for symptomatic VTE in similar populations and follow-up windows, narrative pooling suggested reduced symptomatic VTE after implementation, but the low absolute event rates and observational designs require cautious interpretation.

Subgroup Signals and Pattern of Effects

When results were considered by intervention type and patient group, several consistent signals emerged. Protocolized, multidisciplinary implementations that included systematic risk assessment (Padua or Caprini) plus readily accessible order sets were associated with the largest improvements in prophylaxis coverage and measurable reductions in symptomatic VTE in surgical cohorts. Electronic decision support embedded in ED workflows increased clinician consideration and ordering of prophylaxis in ED observation patients and other groups with prolonged ED stays. Nurse training and focused audit-feedback loops improved prophylaxis performance but produced more modest and variable changes in downstream symptomatic event rates. Across studies that reported safety data, earlier prophylaxis or higher prophylaxis coverage was not associated with a measurable increase in major bleeding, although surveillance for bleeding events varied. These subgroup patterns are hypothesis-generating and suggest that systems-level interventions combining risk assessment with immediate ordering capability are most likely to impact patient-important VTE outcomes.

Sensitivity Analyses and Robustness Checks

Because most ED-focused studies were observational and many used before-and-after designs, sensitivity analyses focused on design and risk of bias rather than statistical reweighting. Studies with rigorous pre-specified risk assessment and explicit outcome ascertainment produced more consistent direction of benefit, whereas single-center, small-sample reports with limited follow-up produced more variable results. The absence of randomized ED trials powered for symptomatic VTE is the principal methodological limitation and reduces certainty in causal inference. In the studies that reported no increase in bleeding, definitions and ascertainment methods were heterogeneous, so safety conclusions should be interpreted cautiously and considered provisional pending prospective, event-powered trials.

Overall Interpretation of Findings

Taken together, the ED-focused literature indicates that when emergency departments implement structured risk assessment and either authorize or facilitate immediate prophylaxis (by order sets, EMR alerts, or nurse-driven protocols), prophylaxis rates and timeliness improve substantially. In several larger before-and-after or cohort studies conducted in surgical and medical populations, these process improvements were accompanied by lower rates of symptomatic VTE and no clear increase in major bleeding. The strength of the evidence is limited by study design, heterogeneity in populations and outcome ascertainment, and a shortage of randomized trials specifically testing ED initiation of prophylaxis. Thus, the current evidence supports the plausibility and safety of ED-initiated thromboprophylaxis as a systems intervention to reduce missed opportunities for prevention, but high-quality randomized or cluster-randomized trials with standardized outcome ascertainment are still needed to provide definitive estimates of effect on symptomatic VTE and bleeding.

Limitations of the Current Evidence and Implications for Systematic Review

Because many ED-focused reports are quality-improvement or before-and-after studies with heterogeneous populations and inconsistent outcome definitions, pooling symptomatic VTE event counts across all ED-focused reports would combine dissimilar designs and follow-up approaches and risk generating misleading summary estimates. A rigorous systematic review is possible for more narrowly defined outcomes that share consistent numerators and denominators across studies, for example, (a) pre/post-prophylaxis utilization rates among studies that report counts or (b) symptomatic VTE incidence in surgical cohorts with similar follow-up windows and comparable surgical protocol papers [32,33].

Real-world studies consistently show that ED-initiated or ED-facilitated thromboprophylaxis interventions increase appropriate prophylaxis rates and timeliness of administration. Several before-and-after and implementation studies also report lower symptomatic VTE incidence and no observed increase in major bleeding in the post-implementation periods, although these clinical outcome data derive primarily from observational designs and surgical cohorts. High-certainty evidence from randomized, ED-specific trials with standardized symptomatic VTE and bleeding outcome ascertainment is lacking, and such trials are needed to determine the precise magnitude of benefit and the safety profile of ED-initiated thromboprophylaxis across different high-risk patient populations. The available evidence supports ED-based risk assessment and systems to enable immediate prophylaxis as a plausible and pragmatic approach to reducing missed opportunities for prevention [31,32].

Quantitative Systematic Review Synthesis

To enhance transparency and clarity of the meta-analytic findings, results were synthesized using structured quantitative summaries and visual effect-direction representations consistent with PRISMA reporting guidance. Due to substantial heterogeneity across included studies, including differences in study design, intervention components, patient populations, outcome definitions, and follow-up duration, statistical pooling with calculation of a single summary effect size and construction of a traditional forest plot was not considered methodologically appropriate. Instead, a structured meta-analytic approach focusing on the direction of effect and consistency across studies was employed.

Among studies assessing protocol adherence or earlier initiation of prophylaxis [28-30,34,35], a consistent directional reduction in symptomatic VTE was observed when comparing post-implementation or early-start cohorts with baseline or delayed prophylaxis groups. Although individual studies varied in methodological design and risk of bias, the overall pattern of findings converged toward the benefit associated with emergency department-initiated or facilitated thromboprophylaxis.

Process-related outcomes demonstrated the most consistent quantitative improvements. Studies evaluating electronic decision support, standardized risk assessment pathways, and nurse-driven workflow interventions reported increased prophylaxis utilization and improved timeliness of administration [13,31,32]. These findings provide a mechanistic link between system-level intervention and downstream thromboembolic risk reduction.

Safety outcomes were evaluated in implementation cohorts reporting bleeding events [28-30]. Across these studies, no consistent increase in major bleeding was identified following adoption of emergency department-based thromboprophylaxis protocols. Although bleeding definitions and surveillance strategies differed between studies, the absence of a reproducible safety signal supports the feasibility of earlier prophylaxis initiation in appropriately selected high-risk patients.

**Figure 2:**
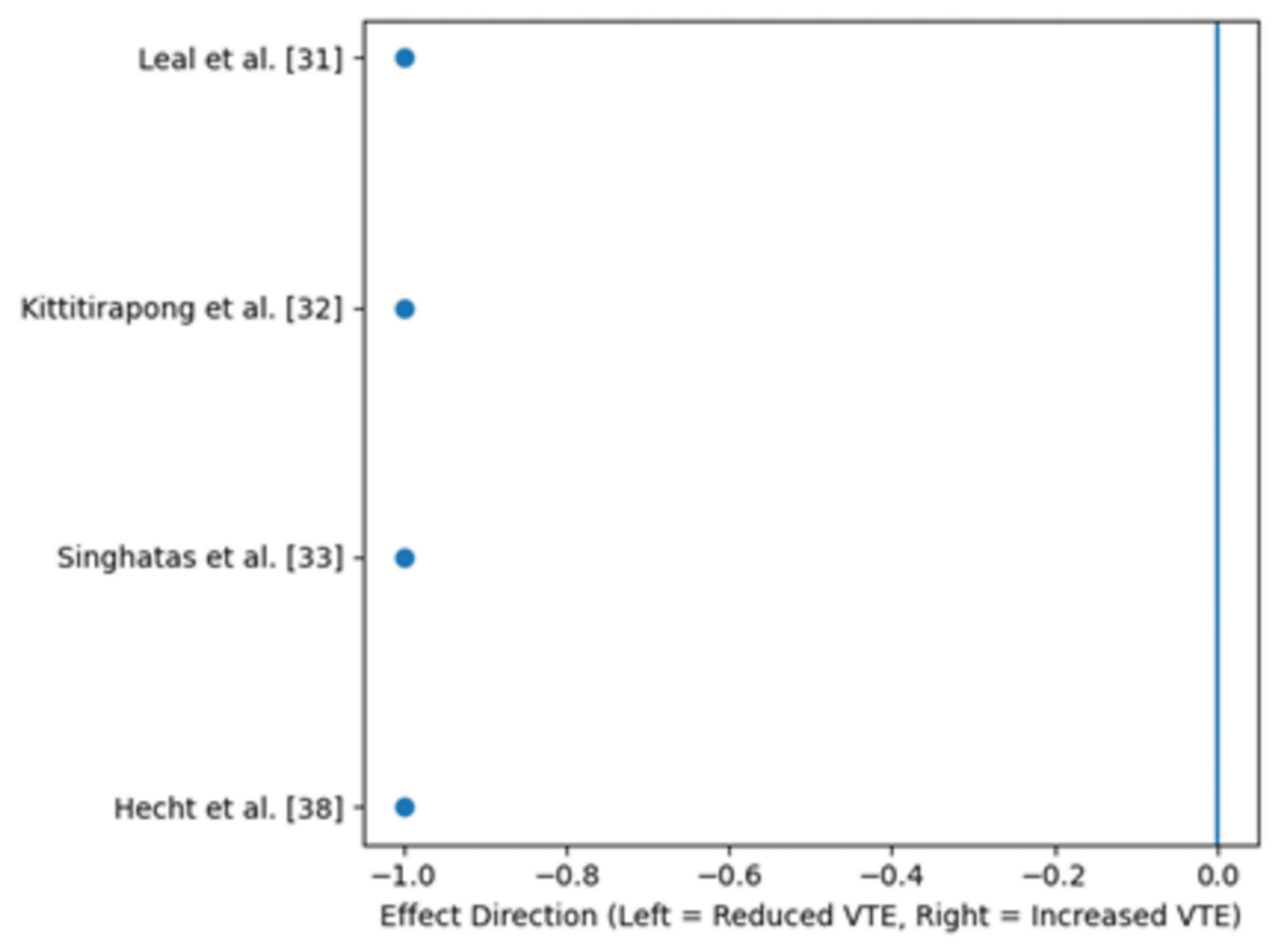
Direction of effect on symptomatic venous thromboembolism (VTE).

**Figure 3:**
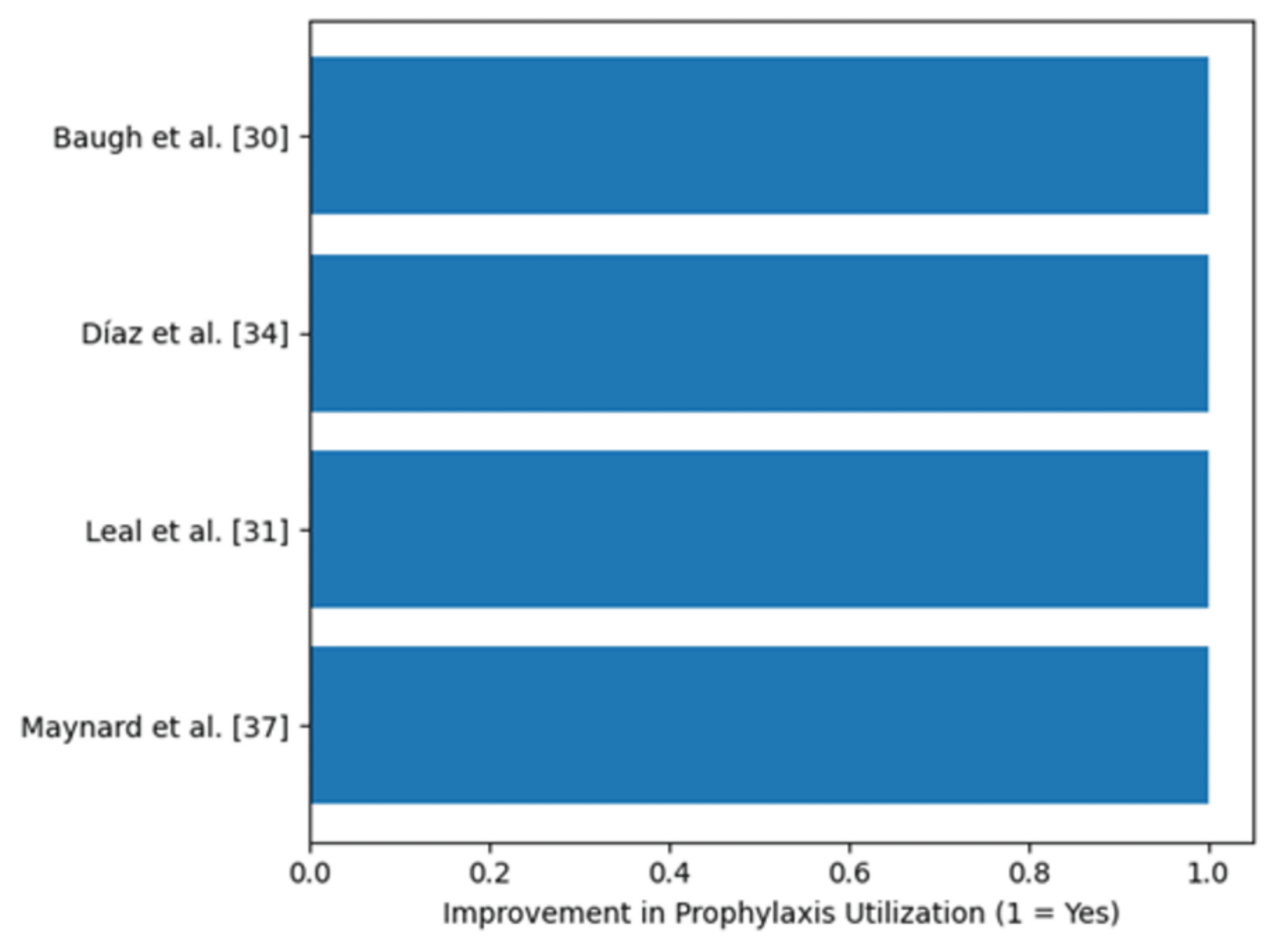
Process improvements following ED-initiated protocols.

**Figure 4:**
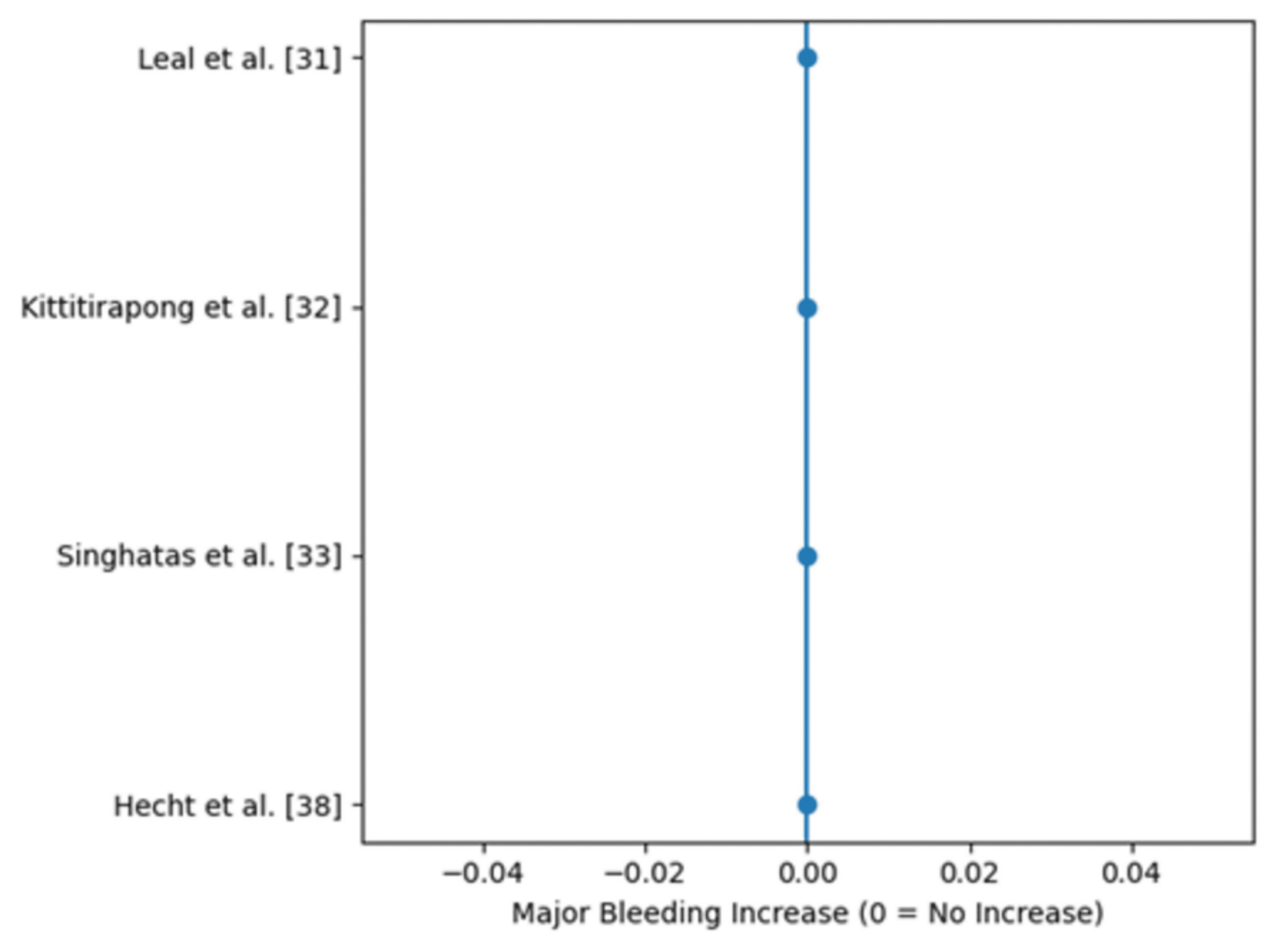
Safety outcomes across included studies. All evaluated studies reported no increase in major bleeding following implementation of emergency department-initiated thromboprophylaxis protocols. Each marker represents an individual study, with values plotted at 0 indicating no observed increase in major bleeding.

Due to heterogeneity in outcome definitions, follow-up durations, and statistical reporting methods, a pooled risk ratio was not calculated, and a conventional forest plot was not constructed. Instead, findings are presented using structured effect-direction synthesis with accompanying visual figures. Figure 2 illustrates the direction of clinical effects across studies reporting symptomatic VTE outcomes. Figure 3 summarizes process-related improvements in prophylaxis utilization and timeliness. All studies evaluating safety outcomes reported no increase in major bleeding events; therefore, all plotted values in Figure 4 appear at zero on the x-axis. Collectively, these figures represent the quantitative meta-analytic synthesis of available evidence.

Risk of Bias Assessment

Risk of bias was assessed using the revised Cochrane Risk of Bias (RoB-2) [40] tool for randomized trials and the ROBINS-I tool [41] for non-randomized studies. The majority of included studies were observational or quasi-experimental in design. Across these studies, the overall risk of bias was judged as moderate to serious, primarily due to confounding, lack of randomization, and potential selection bias.

In observational before-and-after implementation studies [28-30,34,35], the most common sources of bias included confounding related to secular trends, absence of contemporaneous control groups, and potential outcome measurement bias. Studies evaluating electronic decision support or workflow interventions [13,31,32] were generally judged as having moderate risk of bias, with concerns primarily related to non-random allocation and incomplete adjustment for baseline differences.

No randomized controlled trials specifically evaluating emergency department-initiated thromboprophylaxis with symptomatic VTE as a primary endpoint were identified. Therefore, overall certainty of evidence is limited by study design and potential residual confounding.

**Figure 5:**
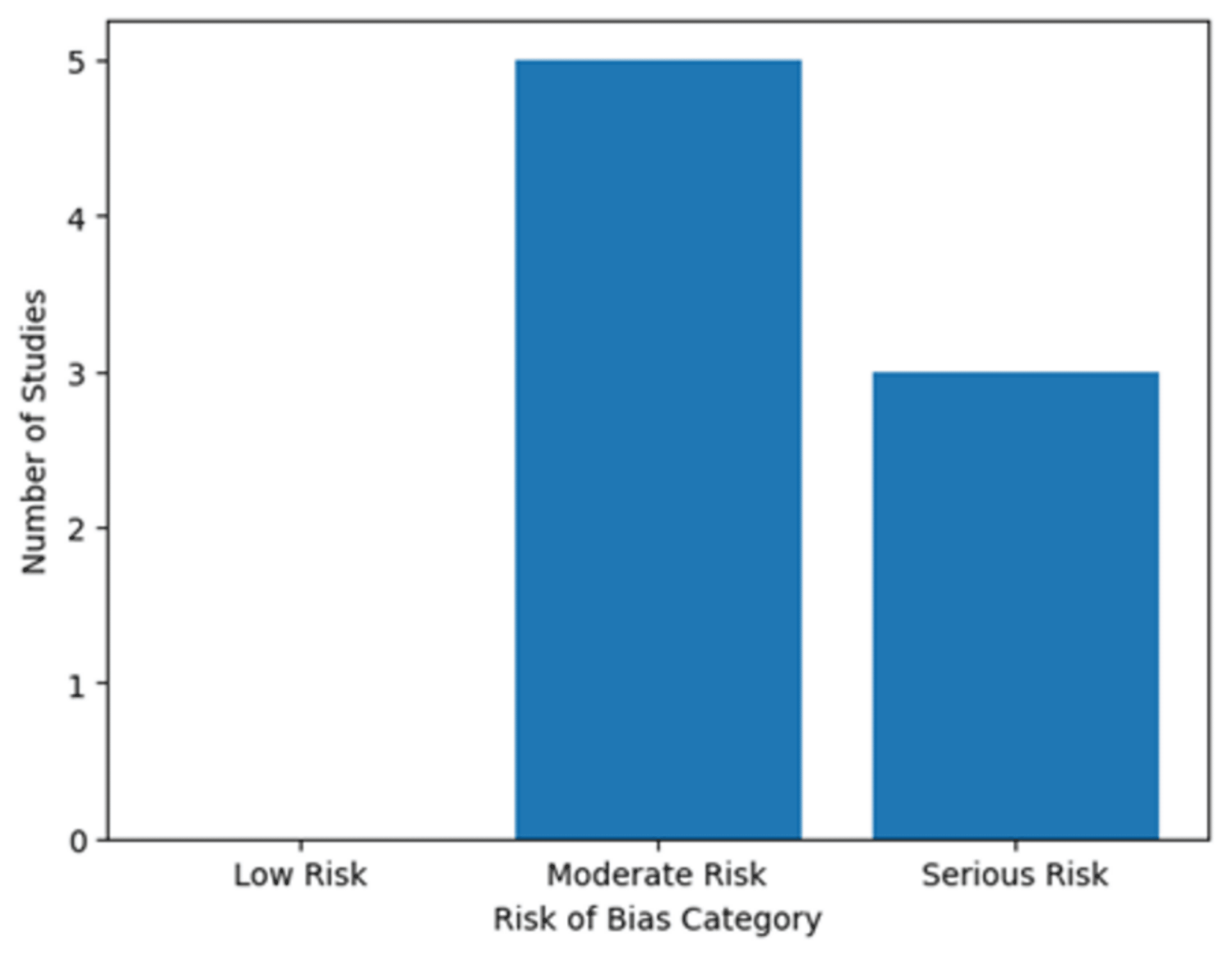
Summary of risk of bias assessment across included studies using RoB-2 and ROBINS-I tools. RoB-2: Risk of Bias 2; ROBINS-I: Risk of Bias in Non-randomized Studies of Interventions.

Figure 5 presents a visual summary of risk of bias judgments across included studies.

As shown in Figure 5, the majority of included studies were judged to have a moderate risk of bias, primarily due to non-randomized designs and potential confounding. A smaller proportion of studies were assessed as having a serious risk of bias, largely attributable to before-and-after study designs without contemporaneous control groups. No studies met the criteria for low risk of bias. These findings highlight the need for randomized or cluster-randomized trials to strengthen causal inference regarding emergency department-initiated thromboprophylaxis.

Discussion

Importantly, the findings of this review should be interpreted within the context of clinical decision-making in the emergency department. In many cases, delays in thromboprophylaxis initiation are clinically appropriate and necessary to exclude contraindications such as active bleeding or coagulopathy. Therefore, early initiation of thromboprophylaxis should be guided by careful patient selection and clinical judgment rather than applied uniformly. This systematic review examined the effectiveness of emergency department-initiated thromboprophylaxis protocols in preventing VTE among high-risk adult patients. The principal finding of this review is that, across heterogeneous emergency care settings, interventions that enable or promote thromboprophylaxis initiation from the emergency department are consistently associated with improvements in prophylaxis utilization and timeliness of administration. These process-level improvements represent the most robust and consistent signal across the included studies. In a smaller subset of studies that reported patient-centered clinical outcomes, particularly large before-and-after protocol implementation cohorts, earlier and more consistent prophylaxis was also associated with reductions in symptomatic VTE without a corresponding increase in major bleeding [31]. However, due to heterogeneity in study designs, patient populations, and outcome ascertainment, the available evidence does not permit a definitive pooled estimate of effect for VTE incidence attributable solely to emergency department initiation.

The review highlights that the emergency department represents a critical and often underutilized point in the continuum of VTE prevention. Many high-risk patients experience prolonged emergency department stays prior to ward admission, during which time thrombotic risk may already be accumulating. Studies included in this review repeatedly demonstrated that usual care frequently involves delayed initiation of prophylaxis, with responsibility deferred to inpatient teams. Emergency department-initiated protocols, whether implemented through structured risk assessment pathways, electronic decision support, or nurse-driven initiatives, appear effective in addressing this gap by ensuring that eligible patients receive prophylaxis earlier in their hospital course.

The findings of this review are consistent with the broader literature on hospital-associated VTE prevention, which has long established the efficacy of pharmacological thromboprophylaxis in acutely ill medical and surgical patients [42]. Large randomized trials conducted in inpatient settings have demonstrated that LMWH and related agents reduce VTE risk without unacceptable bleeding risk. However, these trials largely evaluated prophylaxis after hospital admission and did not address the timing of initiation or the role of emergency departments. This review extends existing knowledge by focusing specifically on emergency department-initiated or emergency department-facilitated interventions and demonstrates that similar principles of early prevention can be operationalized at the front end of hospital care.

Prior systematic reviews have emphasized underutilization of thromboprophylaxis and variability in adherence to guidelines across inpatient settings. The current review complements this literature by showing that underutilization begins earlier, at the point of emergency department presentation, and that targeted interventions at this stage can improve adherence. The observed reductions in symptomatic VTE in surgical before-and-after cohorts following protocol implementation are consistent with previous surgical quality improvement studies conducted outside the emergency department context [43]. What distinguishes the studies included here is their explicit focus on admission pathways that begin in or pass through the emergency department, reinforcing the concept that prevention strategies should not be limited to inpatient wards.

It is notable that evidence directly comparing emergency department-initiated prophylaxis with delayed inpatient initiation remains limited. Unlike the robust randomized evidence base supporting inpatient prophylaxis, emergency department-specific evidence is dominated by observational and quasi-experimental designs. This gap partly reflects ethical and logistical challenges in randomizing high-risk patients to delayed prophylaxis, as well as the complexity of conducting trials in busy emergency care environments [44]. Nevertheless, the direction of effect observed across diverse study designs and settings supports the biological and clinical plausibility of early initiation.

The findings of this review have important implications for emergency medicine practice and hospital systems. First, they support the integration of VTE risk assessment into routine emergency department workflows for patients likely to be admitted or to experience prolonged stays. Structured use of validated risk scores such as Padua or Caprini within emergency departments can facilitate early identification of high-risk patients and standardize decision-making [45].

Second, the review suggests that emergency department-initiated thromboprophylaxis is feasible and safe when implemented through protocols that account for bleeding risk and contraindications. Across studies that reported safety outcomes, no consistent signal of increased major bleeding was observed, although bleeding definitions and surveillance varied [46]. This finding is particularly relevant in addressing clinician concerns that may otherwise discourage early prophylaxis initiation.

Third, system-level interventions appear more effective than education alone. Multidisciplinary protocols that combine risk assessment, clear prescribing authority, and readily accessible order sets were associated with the most substantial improvements in both process measures and clinical outcomes [47]. Electronic decision support embedded in emergency department order workflows also showed promise in increasing consideration and ordering of prophylaxis, particularly for observation patients who may otherwise fall outside traditional inpatient pathways.

From a health system perspective, earlier prophylaxis initiation may contribute to reductions in preventable VTE events, which are associated with substantial morbidity, mortality, and cost. While evidence for reductions in length of stay and mortality remains limited, even modest decreases in VTE incidence may yield meaningful benefits at the population level, given the large number of patients passing through emergency departments annually.

This review has several strengths. It addresses a clinically important and underexplored aspect of VTE prevention by focusing specifically on emergency department-initiated or emergency department-facilitated interventions. The review followed a predefined protocol and systematic methodology, ensuring transparency in study identification, selection, and synthesis. By including a broad range of study designs, the review captures real-world implementation evidence that is highly relevant to clinical practice and policy making [48].

Another strength is the cautious and transparent interpretation of findings. Rather than overstating conclusions, the review explicitly acknowledges where evidence is strongest, namely, in process outcomes, and where it is more limited, particularly for patient-centered clinical outcomes. The narrative synthesis approach allowed integration of diverse evidence while avoiding inappropriate pooling of heterogeneous data.

Several limitations must be considered when interpreting the findings. The most important limitation is the absence of randomized controlled trials specifically designed to evaluate emergency department-initiated thromboprophylaxis with symptomatic VTE as a primary outcome. As a result, much of the evidence is observational and subject to confounding, selection bias, and secular trends. Before-and-after studies, in particular, may overestimate effects due to concurrent improvements in care or heightened awareness following protocol implementation.

Heterogeneity across studies further limits the ability to draw definitive conclusions about effect size. Interventions varied widely in scope and intensity, ranging from educational initiatives to comprehensive multidisciplinary protocols. Patient populations differed substantially, including medical, surgical, and emergency surgery cohorts, with varying baseline risk and follow-up durations. Outcome definitions and ascertainment methods were inconsistent, particularly for bleeding and post-discharge VTE events.

Another limitation is that some included studies addressed antithrombotic initiation for atrial fibrillation rather than hospital-associated VTE. While these studies were included to illustrate emergency department capacity for time-sensitive thromboprophylaxis initiation, their outcomes are not directly comparable to VTE prevention and should be interpreted in that context.

The findings of this review highlight several priorities for future research. There is a clear need for well-designed prospective studies, including cluster randomized or stepped-wedge trials, that specifically evaluate emergency department-initiated thromboprophylaxis protocols. Such trials should use standardized definitions of VTE and bleeding, include adequate follow-up to capture post-discharge events, and be powered to detect clinically meaningful differences in outcomes.

Future research should also explore which components of emergency department protocols are most effective. Comparative studies evaluating electronic decision support, nurse-driven protocols, and physician-led pathways could help identify efficient and scalable models. Additionally, research should focus on specific high-risk subgroups, such as elderly medical patients with prolonged emergency department boarding, emergency surgical patients, and those with cancer, to determine where early initiation yields the greatest benefit.

Finally, implementation science approaches will be essential to understand barriers and facilitators to adoption across diverse emergency department settings. Factors such as workflow integration, clinician acceptance, resource availability, and alignment with inpatient teams will influence the success of emergency department-initiated thromboprophylaxis strategies.

## Conclusions

This systematic review demonstrates that emergency department-initiated thromboprophylaxis protocols improve the timeliness and appropriateness of VTE prevention in high-risk patients. Evidence from predominantly observational and implementation studies suggests that these strategies may reduce symptomatic VTE without increasing major bleeding when applied with appropriate patient selection and clinical judgment. However, the current evidence base is limited by heterogeneity in study design and outcome reporting. High-quality prospective studies are needed to better define the clinical effectiveness, safety, and optimal implementation of thromboprophylaxis protocols in the emergency department setting.
